# Dietary hempseed and cardiovascular health: nutritional composition, mechanisms and comparison with other seeds

**DOI:** 10.3389/fnut.2025.1669375

**Published:** 2025-10-08

**Authors:** Ömer Furkan Kaçar, Tugba Kose, Hüsna Kaya Kaçar

**Affiliations:** ^1^Doctoral School of Health Sciences, Faculty of Health Sciences, University of Pécs, Pécs, Hungary; ^2^Department of Biochemistry and Medical Chemistry, Medical School, University of Pécs, Pécs, Hungary; ^3^Nutrition and Dietetics Department, Sabuncuoglu Serefeddin Education and Research Hospital, Amasya University, Amasya, Türkiye; ^4^Food Innovation and Health, Quadram Institute Bioscience, Norwich Research Park, Norwich, United Kingdom; ^5^Department of Nutritional Sciences, School of Life Course and Population Sciences, King’s College London, London, United Kingdom; ^6^Department of Nutrition and Dietetics, Faculty of Health Sciences, Hitit University, Çorum, Türkiye; ^7^Nutrition and Food Research Center, Faculty of Medicine, University of Turku, Turku, Finland; ^8^Division of Nutrition and Dietetics, Faculty of Health Sciences, Amasya University, Amasya, Türkiye

**Keywords:** hempseed, *Cannabis sativa* L., cardiovascular disease, functional food, inflammation, oxidative stress

## Abstract

Cardiovascular disease (CVD) remains the leading cause of global mortality, with dietary habits playing a significant role in its prevention and management. Hempseed (*Cannabis sativa* L.) has gained recognition as a functional food due to its rich nutritional profile, including high-quality plant proteins, optimal omega-6 to omega-3 fatty acid ratios, and a variety of bioactive compounds such as tocopherols, phytosterols, and polyphenols. This review critically evaluates the potential cardioprotective effects of hempseed, focusing on its impact on lipid metabolism, inflammation, oxidative stress, and other cardiometabolic markers. Preclinical studies suggest that hempseed can improve lipid profiles, reduce blood pressure, and reduce oxidative stress and inflammation, though clinical evidence remains limited and findings from animal models may not directly translate to human cardiovascular benefits due to physiological differences between species. This review further evaluates hempseed’s potential in cardiovascular disease prevention and highlights its potential advantages when compared with other widely consumed seeds (flaxseed and chia seeds), emphasizing its unique fatty acid composition, optimal omega-6 to omega-3 ratio, and diverse bioactive compounds. Despite the promising findings, there is a need for long-term randomized controlled trials to establish the efficacy and safety of hempseed in diverse populations. This review emphasizes the potential of hempseed as a dietary intervention for CVD prevention and calls for further research to optimize its use in clinical and public health settings.

## Graphical abstract

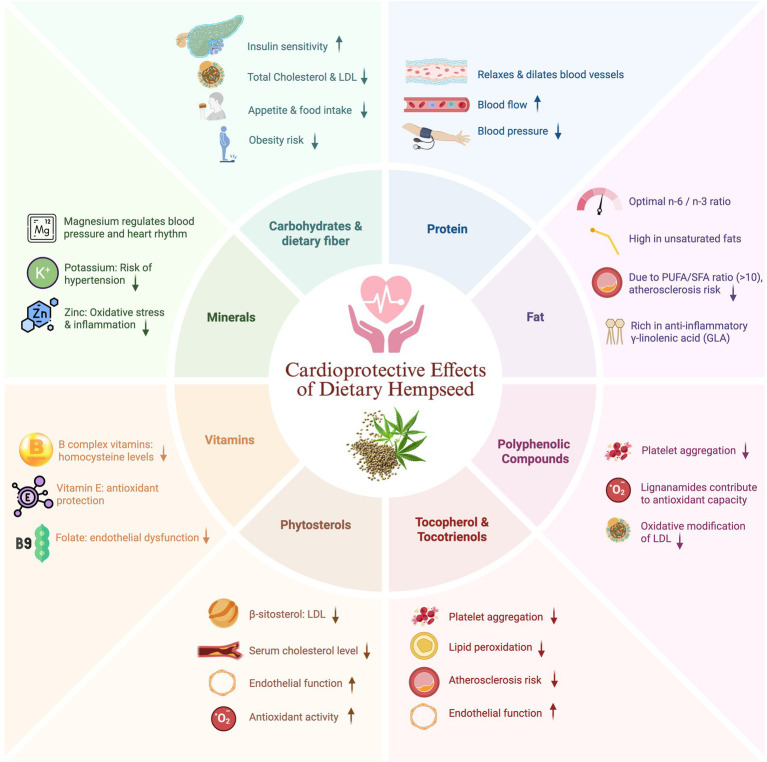
 Created with BioRender.com.

## 1 Introduction

Cardiovascular disease (CVD) remains the leading cause of mortality worldwide and comprises a broad spectrum of disorders affecting the heart and blood vessels, including coronary artery disease, stroke, heart failure, and peripheral artery disease (1, 2). According to recent global estimates, the prevalence of CVD is linked to approximately 17.9 million annual deaths, with ischemic heart disease and stroke representing the leading contributors (3–5). Cardiometabolic disorders (CMD), a cluster of interrelated conditions including obesity, type 2 diabetes mellitus (T2DM), dyslipidemia, and hypertension, significantly elevate the risk of developing CVD through shared pathophysiological mechanisms (6). These mechanisms include insulin resistance, chronic low-grade inflammation, endothelial dysfunction, and oxidative stress, all of which contribute to accelerated atherosclerosis and increased cardiovascular risk (7, 8).

Nutrition is a cornerstone in the prevention and management of CVD and CMD, with compelling evidence from epidemiological studies and clinical trials demonstrating the profound impact of dietary patterns on cardiometabolic risk factors (9, 10). Unhealthy dietary habits, characterized by excessive consumption of refined carbohydrates, saturated fats, and ultra-processed foods, contribute significantly to the development of obesity, dyslipidemia, insulin resistance, and hypertension (11, 12). Conversely, dietary patterns rich in whole foods, fiber, unsaturated fats, and bioactive compounds have been associated with improved lipid profiles, reduced blood pressure, and enhanced glycemic control (13). The protective effects of plant-based diets against CVD and CMD are attributed to their high dietary fiber, polyphenol, unsaturated fatty acid, and essential micronutrient contents (14, 15).

Functional foods, defined as foods or food components that offer physiological advantages or reduce disease risk beyond basic nutrition, have gained significant attention in cardiovascular health research (16). These foods typically contain bioactive compounds, including polyunsaturated fatty acids (PUFAs), dietary fiber, polyphenols, and phytosterols, which positively influence cardiometabolic markers (14, 17). Notable examples with demonstrated cardiovascular benefits include fatty fish rich in omega-3 fatty acids, nuts, seeds high in unsaturated fats and antioxidants, and whole grains, which contribute to improved lipid metabolism and reduced inflammation (18, 19). Plant-based functional foods, in particular, are increasingly being recognized for their potential in preventing CVD while minimizing the harmful effects associated with excessive animal-based food consumption (20).

Hempseed refers to the edible seeds produced by the plant *Cannabis sativa* L. With a history of cultivation spanning thousands of years across various cultures, from ancient China to Mesopotamia, hemp has long served as a source of fiber, oil, and food (21, 22). Unlike its psychoactive counterpart, marijuana, industrial hempseed contains negligible tetrahydrocannabinol (THC) levels (<0.3%), making it a safe and legal dietary component worldwide (23). The nutritional profile of hempseed is particularly noteworthy, featuring high-quality plant proteins containing all essential amino acids in ratios comparable to those of animal-based proteins (24). Hempseed’s cardiovascular benefits are potentially derived from its optimal 3:1 omega-6: omega-3 fatty acid ratio and rich bioactive profile (including *γ*-linolenic acid, tocopherols, phytosterols, and polyphenols) (22, 24). Preclinical evidence suggests multiple cardioprotective mechanisms, such as improved lipid profiles, antihypertensive effects, reduced cholesterol absorption, and attenuated oxidative stress and inflammation. However, it is important to note that while preclinical studies show promise, the clinical evidence base remains limited and inconclusive, with some studies reporting conflicting results regarding hempseed’s cardiovascular effects.

Given the substantial global burden of CVD and CMD, coupled with the growing emphasis on dietary strategies for prevention, elucidating the potential of functional foods such as hempseed represents an important scientific and public health priority. This comprehensive review aims to critically evaluate the existing evidence regarding the impact of hempseed on CVD and CMD risk factors, with a particular focus on its effects on lipid metabolism, inflammation, oxidative stress, and other relevant cardiometabolic markers. Additionally, we sought to clarify the underlying mechanisms through which hempseed, and its bioactive components exert their potential cardioprotective effects and critically compare them with other commonly consumed seeds, namely flaxseed and chia seed, from a nutritional perspective. By synthesizing the current findings and identifying research gaps, this review provides a foundation for future clinical investigations and evidence-based dietary recommendations incorporating hempseed for cardiometabolic health promotion.

## 2 Methods

A comprehensive literature search was conducted using PubMed, Scopus, Web of Science, and Cochrane Library databases from inception through April 2025. The search strategy employed a combination of MeSH terms and keywords including: (“*Cannabis sativa*” OR “hempseed” OR “hemp seed”) AND (“cardiovascular” OR “cardioprotective” OR “heart disease” OR “lipid” OR “cholesterol” OR “hypertension” OR “inflammation” OR “oxidative stress”). Boolean operators (AND, OR) were used to combine search terms effectively. Reference lists of included articles were manually screened for additional relevant studies.

Articles published within the last two decades were prioritized to ensure inclusion of recent findings; however, earlier studies were also included to provide critical background. Studies were included if they: (1) investigated hempseed or its components in relation to cardiovascular health outcomes; (2) included preclinical (animal models) or clinical studies; (3) measured relevant biomarkers (lipid profiles, inflammatory markers, blood pressure, vascular function); and (4) were published in peer-reviewed journals. Reviews and meta-analyses were used to contextualize the evidence, summarize consensus, highlight discrepancies, and identify gaps in knowledge. Exclusion criteria included: conference abstracts, studies focusing only on cannabis psychoactive compounds, and studies without cardiovascular-related outcomes. Also, duplicate articles, non-English publications, and articles without full text availability were excluded during screening.

The authors declare that they have no financial or non-financial conflicts of interest that have influenced the selection of research articles, or the outcomes and interpretations presented in this review.

## 3 Nutritional composition of hempseed

**Figure 1:**
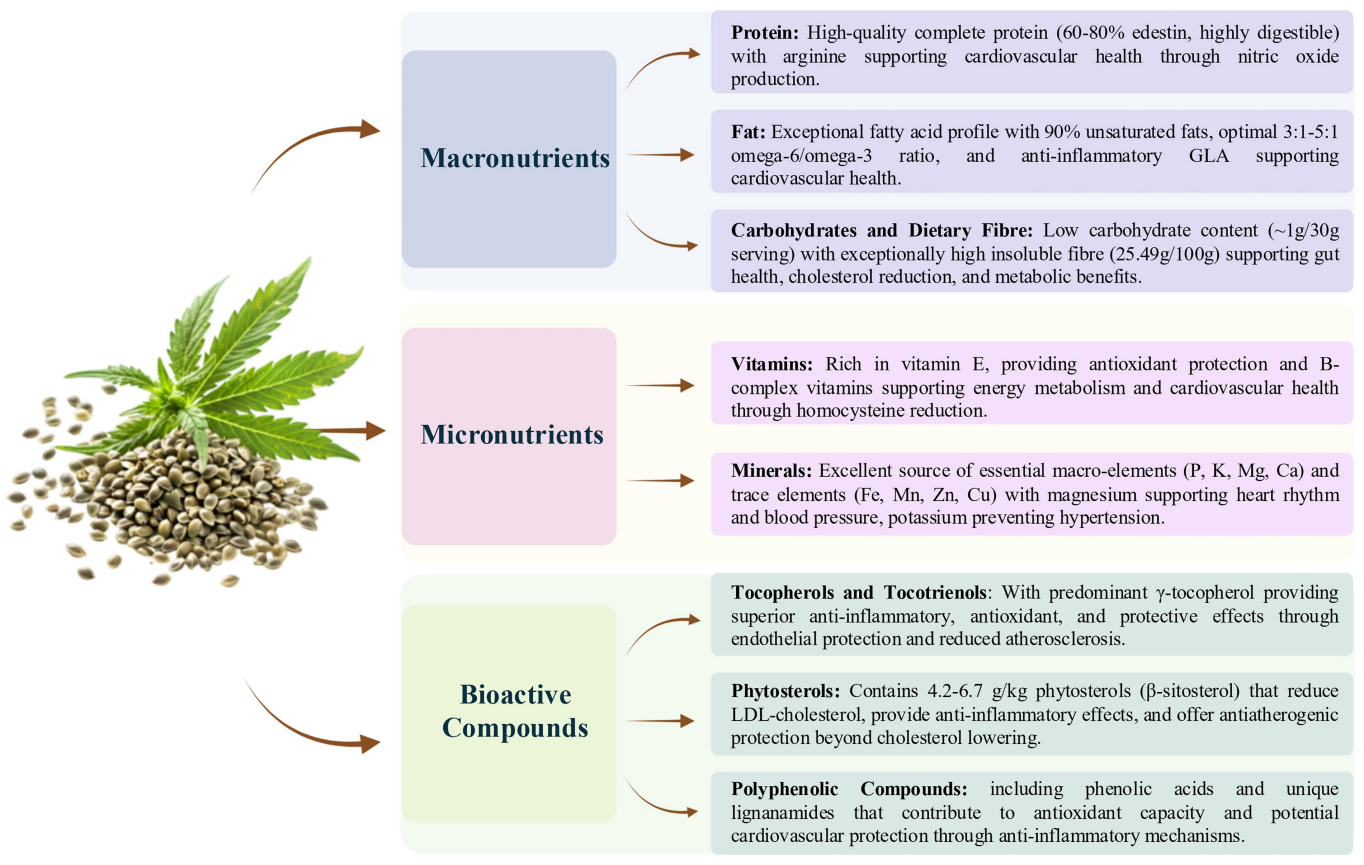
A summary of the nutritional composition of hempseed and its impact on cardiovascular health.

Hempseed, the edible fruit of *Cannabis sativa* L., was historically viewed just as a by-product of hemp fiber production (25). However, with the recent revival of industrial hemp cultivation (varieties containing less than 0.3% or 0.2% *Δ*-9 THC), this seed has gained significant attention because of its exceptional nutritional profile and functional properties (26). The growing interest in hempseed production originates from its remarkable nutrient composition and potential health benefits compared to other edible seeds. Indeed, hempseeds are recognized for their balanced and nutrient-dense macronutrient profiles, which contribute to their appeal as functional foods (27). A typical 30 g serving of hempseeds contains approximately 170 calories, with macronutrients distributed as follows: 10 g of protein, 14 g of fat, and 1 g of carbohydrates (24, 28). This composition makes hempseeds a rich source of plant-based protein and essential fatty acids (EFAs), particularly omega-3 and omega-6, in an optimal ratio, offering potential health benefits for cardiovascular health through improved lipid profiles, reduced inflammation, and enhanced vascular function (22, 29). Figure 1 provides an overview of the nutritional composition of hempseed and its potential effects on cardiovascular health.

### 3.1 Macronutrients

#### 3.1.1 Protein

Hempseed is a high-quality plant-based protein source that contains all nine essential amino acids required for human health. Protein quality depends on the amino acid profile, digestibility, and bioavailability criteria of hempseed protein. The protein content is primarily composed of edestin (60–80%) and albumin, which are both highly digestible and bioavailable (30). Glutamic acid is the most abundant amino acid (3.74–4.58% of the whole seed), followed by arginine (2.28–3.10% of the whole seed). It is important to note that arginine serves as a precursor of nitric oxide (NO), which relaxes and dilates blood vessels, potentially improving vascular health by enhancing blood flow, reducing blood pressure, and improving overall cardiovascular health. Hempseed protein demonstrates digestibility comparable to other plant-based protein sources, such as soy, making it an excellent alternative for plant-based nutrition (31).

#### 3.1.2 Fat

Hempseed oil has an optimal fatty acid profile, containing up to 90% unsaturated fatty acids, with PUFAs comprising 70–80% of this fraction (32). The predominant monounsaturated fatty acid is oleic acid (OA, 18:1, n-9), whereas the most abundant PUFAs are linoleic acid (LA, 18:2, n-6) and *α*-linolenic acid (ALA, 18:3, n-3) (24, 32). These EFAs cannot be synthesized endogenously in mammals and serve as precursors to biologically active long-chain PUFAs, which are converted to arachidonic acid (AA, 20:4, n-6), while ALA is transformed into docosahexaenoic acid (DHA, 22:6, n-3) and eicosapentaenoic acid (EPA, 20:5, n-3) (33). These conversions are crucial for maintaining cell membrane structure, cardiovascular health, inflammatory regulation, skin integrity, and neurological function.

The n-6/n-3 ratio in hempseed oil ranges from 3:1 to 5:1, which corresponds with the European Food and Safety Authority’s recommendations for health maintenance and chronic disease prevention (21). This balanced ratio mirrors the traditional Japanese and Mediterranean diets associated with low coronary disease incidence, contrasting sharply with the typical Western diets that feature an unhealthy 10:1 ratio (34). While controversy exists regarding optimal omega-6 to omega-3 ratios, with some studies suggesting ratios as low as 1:1 may be ideal for reducing inflammatory responses, the current evidence indicates that ratios between 2:1 to 4:1 provide cardiovascular benefits compared to the typical Western diet ratio of 10–15:1 (35, 36). The debate continues regarding whether absolute intake of these fatty acids or their relative ratios is more important for cardiovascular outcomes, though both factors likely contribute to overall inflammatory status (37).

Hempseed oil also contains *γ*-linolenic acid (GLA, 18:3, n-6) and stearidonic acid (SDA, 18:4, n-3), which bypass the rate-limiting *δ*-6-desaturase enzyme and facilitate the conversion to biologically active long-chain PUFAs. GLA specifically exhibits potent anti-inflammatory properties (38). Unlike other GLA-rich sources, such as borage oil (19–23% GLA), hempseed oil provides the added benefit of n-3 PUFAs. The saturated fatty acid content remains below 12%, primarily comprising palmitic acid (PA, 16:0, 2–9%) and stearic acid (SA, 18:0), providing a favorable PUFA/SFA ratio (>10) associated with reduced atherosclerosis and coronary heart disease risk (39).

#### 3.1.3 Carbohydrates and dietary fiber

Hempseed contains minimal carbohydrates (approximately 1 g per 30 g serving), making it suitable for low-carbohydrate diet. The total carbohydrate content typically ranges from 20 to 30% and is predominantly composed of dietary fiber with both soluble and insoluble fractions. Research has shown that hempseed contains high levels of insoluble dietary fiber (25.49 g/100 g), whereas soluble dietary fiber content is significantly lower (0.16 g/100 g), positioning hempseed as one of the richest sources of IDF among high-protein crops (21, 40).

Although a high fiber content may slightly reduce protein digestibility, the health benefits of dietary fiber are substantial. Fiber functions as a prebiotic, enhances insulin sensitivity, reduces appetite and food intake (lowering obesity and diabetes risk), and decreases total cholesterol and low-density lipoprotein (LDL) levels. Furthermore, dietary fiber resists digestion in the small intestine and reaches the large intestine, where the gut microbiota ferments it into short-chain fatty acids with anti-inflammatory properties, further enhancing the role of hempseed in supporting metabolic health (41, 42).

### 3.2 Micronutrients

#### 3.2.1 Vitamins

Hempseed contains a variety of vitamins, including significant levels of vitamin E and B-complex vitamins. Vitamin E, in particular, is a potent antioxidant that protects cells from oxidative stress, which is a key factor in the development of CVDs (43). Hempseed oil is a particularly rich source of tocopherols, the most active form of vitamin E, which has been shown to have protective effects on blood vessels and reduce the risk of atherosclerosis (23).

Hempseed also contains several B-vitamins, including B1 (thiamine), B2 (riboflavin), B3 (niacin), B6 (pyridoxine), and folate, which are essential for energy metabolism, red blood cell production, and maintenance of healthy nerve function (31). These vitamins contribute significantly to the reduction in homocysteine levels, which is a well-established risk factor for CVD (44, 45).

#### 3.2.2 Minerals

Based on the Dietary Reference Value of the European Food Safety Authority’s Dietary Reference Value (DRV) and the Institute of Medicine’s Dietary Reference Intake (DRI), hempseed is an excellent natural source of key macro-elements including phosphorus (P), potassium (K), magnesium (Mg), calcium (Ca), and sodium (Na). Additionally, it contains important trace elements such as iron (Fe), manganese (Mn), zinc (Zn), and copper (Cu) (46).

Magnesium deserves particular attention in the context of cardiovascular health. It is involved in over 300 enzymatic reactions in the body and is essential for maintaining normal heart rhythm, muscle function, and blood pressure regulation (47). Magnesium deficiency has been linked to an increased risk of hypertension and CVD, making it an important mineral for heart health.

Potassium, another key mineral abundant in hempseed, helps maintain proper electrolyte balance, regulates fluid levels, and reduces the risk of hypertension by promoting vasodilation (48). Therefore, potassium is an essential component in the prevention and management of CVDs.

Zinc, a trace element with antioxidant properties found in hempseed, contributes to immune function, protein synthesis, and wound healing. It also plays a significant role in reducing oxidative stress and inflammation, both of which have been implicated in the pathogenesis of CVDs (49).

### 3.3 Bioactive compounds

#### 3.3.1 Tocopherols and tocotrienols

Hempseed contains high concentrations of vitamin E compounds (tocopherols and tocotrienols), which are potent lipophilic antioxidants that may confer cardioprotective benefits. The total tocopherol content in hempseed ranges from to 90–150 mg/100 g, predominantly composed of *γ*-tocopherol (approximately 85% of the total tocopherol content), followed by *α*-tocopherol, *δ*-tocopherol, and *β*-tocopherol in descending order of concentration (21, 50). This distribution pattern distinguishes hempseed from many other oilseeds that typically contain higher proportions of α-tocopherol.

The presence of *γ*-tocopherol in hempseed is significantly important when considering cardiovascular health. Although α-tocopherol has traditionally received more attention, emerging evidence suggests that *γ*-tocopherol may possess superior anti-inflammatory and antioxidant properties in vascular tissues (51). Mechanistically, γ-tocopherol effectively neutralizes reactive nitrogen species and peroxynitrite, which are implicated in endothelial dysfunction and atherosclerosis pathogenesis (52, 53). Additionally, *γ*-tocopherol has demonstrated the capacity inhibit cyclooxygenase-2 (COX-2) activity, thereby reducing proinflammatory prostaglandin E2 synthesis in vascular tissues (54).

In experimental models, dietary supplementation with *γ*-tocopherol-rich oils has been associated with reduced platelet aggregation, improved endothelial function, and attenuation of lipid peroxidation (55). Collectively, these effects may contribute to reduced atherosclerotic plaque formation and improved vascular homeostasis (56). Therefore, the rich γ-tocopherol content of hempseed represents a potentially valuable attribute for cardiovascular risk-reduction strategies.

#### 3.3.2 Phytosterols

Hempseed is rich in phytosterols, which are plant-based compounds that are structurally similar to cholesterol. These compounds can compete with cholesterol for absorption in the intestine, potentially leading to a decrease in blood cholesterol levels. The total phytosterol content of hempseed ranges from 4.2–6.7 g/kg, with *β*-sitosterol (67–70% of total phytosterols), campesterol (15–17%), and stigmasterol (2–4%) representing the predominant sterols (57, 58).

The hypocholesterolemic effects of phytosterols have been well documented in numerous clinical trials, with a recent meta-analysis indicating that the daily consumption of 2–3 g of plant sterols can reduce LDL-cholesterol by 8–15% (59). In addition to their cholesterol-lowering properties, phytosterols exhibit pleiotropic effects that may benefit cardiovascular health, including anti-inflammatory actions, improved endothelial function, and antioxidant activities (60).

*β*-Sitosterol, the predominant phytosterol in hempseed, has demonstrated particularly promising cardioprotective properties. In experimental models, β-sitosterol treatment reduced the expression of vascular cell adhesion molecule 1 (VCAM-1) and intercellular adhesion molecule 1 (ICAM-1) in endothelial cells, attenuated foam cell formation, and modulated the inflammatory response of macrophages (61, 62). These effects suggest potential antiatherogenic properties that extend beyond cholesterol reduction alone.

Therefore, the phytosterol content of hempseed, particularly when consumed as part of a balanced diet, may contribute to improved lipid profiles and reduced cardiovascular risk via multiple mechanisms.

#### 3.3.3 Polyphenolic compounds

Although hempseed contains a more modest polyphenolic profile compared to some other seeds, its phenolic compounds nonetheless contribute to its overall antioxidant capacity and potential cardioprotective properties. The total phenolic content of hempseed ranges from 2.2–3.7 mg GAE/g (gallic acid equivalents), with phenolic acids representing the major class of phenolic compounds (63, 64).

The predominant phenolic acids identified in hempseed include p-hydroxybenzoic, vanillic, caffeic, chlorogenic, and ferulic acids (65). These compounds demonstrated varying degrees of radical-scavenging activity and may contribute to the overall antioxidant profile of hempseed. Additionally, lignanamides, which are unique phenolic compounds formed from the condensation of phenylpropanoids with amines, have been identified in hempseed, including cannabisin A, B, and D, as well as grossamide (66).

Despite their relatively low concentrations compared to other bioactive compounds in hempseed, these phenolic constituents may still confer cardiovascular benefits. Polyphenolic compounds have demonstrated capacity to modulate endothelial NO production, inhibit platelet aggregation, reduce the expression of proinflammatory cytokines, and attenuate the oxidative modification of LDL particles—all processes implicated in the pathogenesis of atherosclerosis (67, 68).

Therefore, the anti-inflammatory and antioxidant properties of hempseed polyphenols may contribute to a reduction in oxidative stress and inflammatory processes that underlie CVD development and progression.

## 4 Mechanisms of action in cardiovascular health

### 4.1 Anti-inflammatory properties

Inflammation plays a crucial role in the development and progression of CVDs (69, 70). Chronic low-grade inflammation is a key factor in the initiation and progression of CVDs (69, 71), and is characterized by the upregulation of circulating pro-inflammatory cytokines, including tumor necrosis factor (TNF), interleukin6 (IL-6) and interleukin-1 beta (IL-1β) (72, 73). Inflammation is involved in all stages of atherosclerotic plaque formation, including the initiation of atherogenic processes, in which endothelial cells are activated by oxidized lipoproteins, leading to the expression and release of inflammatory cytokines (73). Inflammation also participates in the late stage of atherosclerosis by enhancing local accumulation of macrophages, which weakens the fibrous lining of atherogenic plaques by secreting collagen-degrading matrix metalloproteinases (MMPs) (73, 74). Furthermore, inflammation is linked to the development of arrhythmias, and CVD prognosis has been shown to be correlated with the levels of inflammatory markers such as ICAM-1, interleukin-6, C-reactive protein (CRP), and serum amyloid-A (72, 75).

The components of hempseed, including lignanamides, terpenes, fatty acids, and Glaucocalyxin A (GLA), have been shown to have anti-inflammatory properties (23, 32, 76). Lignanamides in hempseed have been shown to have anti-inflammatory properties, particularly in reducing the production of pro-inflammatory cytokines, such as IL-6, TNF-*α*, and IL-1β (66, 77). These compounds inhibit the nuclear factor kappa B (NF-κB) inflammatory pathway, which is involved in the regulation of inflammatory responses (78). Lignanamides, such as cannabisin F, grossamide, and coumaroylaminobutanol glucopyranoside (CLG), have been shown to decrease the release of inflammatory cytokines in lipopolysaccharide (LPS)-stimulated BV-2 microglial cells (24, 78, 79). Additionally, these compounds have been found to promote the expression of nuclear factor erythroid 2-related factor 2 (Nfr-2), an important protein that modulates redox homeostasis and regulates the cellular antioxidant response against reactive oxygen species (ROS) (24, 80). The effects of hemp seed alone and combined with aerobic exercise in young adults were evaluated in a recent study and found that hempseed supplementation improved metabolic markers and oxidative stress (81). By upregulating the Nfr-2 pathway, lignanamides provide beneficial antioxidant effects and reduce oxidative stress (82, 83). Overall, the anti-inflammatory properties of lignanamides in hempseed highlight their potential for preventing various pathological conditions, including cardiovascular disorders (84).

Studies have identified several terpenes in hempseed, including *β*-myrcene and *β*-caryophyllene, which have anti-inflammatory effects (85–87). One of the mechanisms by which terpenes reduce inflammation is by decreasing the production of pro-inflammatory cytokines, such as TNF-*α* and IL-1β (88, 89). β-caryophyllene, a sesquiterpene found in hempseed, has been shown to bind to the cannabinoid receptors type 2 (CB2), which can lead to anti-inflammatory effects (89). Additionally, terpenes such as β-myrcene have been found to possess anti-inflammatory, analgesic, and anxiolytic properties (85). Furthermore, terpenes can modulate the expression of genes involved in inflammation, such as inducible nitric oxide synthase (iNOS) and COX-2 (90). The anti-inflammatory effects of terpenes in hempseed can be attributed to their ability to interact with neurotransmitter receptors and modulate the signaling pathways involved in inflammation (85).

Specific fatty acids found in hempseed have anti-inflammatory properties (32). The omega-6 and omega-3 fatty acids in hempseed oil have been shown to have different effects on inflammation. Omega-6 fatty acids, such as LA, can promote inflammation, whereas omega-3 fatty acids, such as ALA, can reduce inflammation (91). Oleic acid has been shown to reduce inflammation by inhibiting the production of pro-inflammatory cytokines and eicosanoids (24). The ratio of omega-6 to omega-3 fatty acids in hempseed oil is approximately 3:1, which is considered a healthy ratio (21). The combination of these fatty acids in hempseed oil may have synergistic anti-inflammatory effects (24). A recent case report concluded that, because of its composition, hemp seed oil may serve as an anti-inflammatory agent, offering notable benefits either on its own or in combination with medications (92). The anti-inflammatory effects of hempseed fatty acids are also related to their ability to reduce the production of pro-inflammatory cytokines, such as TNF-*α*, IL-6, and IL-1β (93). A study on growing female C57BL/6 mice found that dietary supplementation with hempseed oil reduced the serum levels of these cytokines and improved bone parameters and body composition (94).

GLA is a compound found in hempseed oil that has been shown to reduce inflammation (24, 95). GLA found in hempseed has ability to modulate various signaling pathways, including the NF-κB pathway (15). In addition, GLA reduces inflammation by inhibiting the production of pro-inflammatory cytokines such as, IL-1β, IL-6, and TNF-*α* (15). GLA has been shown to decrease the expression of these cytokines in various cell types, including human renal proximal tubular epithelial cells (HK-2) (96). Another mechanism by which GLA reduces inflammation is the activation of the Akt/Nrf2/HO-1 signaling pathway, which is involved in antioxidant responses and cell protection (96, 97). By activating this pathway, GLA increases the expression of antioxidant enzymes, such as superoxide dismutase (SOD) and haeme oxygenase-1 (HO-1), which help reduce oxidative stress and inflammation. GLA also exerts anti-inflammatory effects by inhibiting the production of leukotriene B4 (LTB4), a pro-inflammatory mediator produced by polymorphonuclear neutrophils (PMN) (24). By increasing the plasma levels of its biological metabolite DGLA, GLA can directly suppress the production of LTB4, leading to reduced inflammation (98).

### 4.2 Antioxidant effects

The overall antioxidant capacity of hempseed reflects the cumulative and potentially synergistic effects of its diverse bioactive compounds, including tocopherols, phytosterols, and phenolic compounds. The oxygen radical absorbance capacity (ORAC) of hempseed ranges from to 18–26 μmol TE/g (Trolox equivalents), which is a moderate antioxidant food source (21, 99).

Hempseed is recognized for its high content of phenolic compounds, particularly hydroxycinnamic acid amides and lignanamides, which significantly contribute to its antioxidant properties (100, 101). These compounds have been found to have strong antioxidant activity, which can be attributed to their ability to scavenge free radicals and chelate metal ions (101). Studies have shown that hempseed oil and hempseed protein extracts have antioxidant activity, as measured by various methods, such as DPPH (2,2-diphenyl-1-picrylhydrazyl) radical scavenging activity (101–104). The antioxidant activity of hempseed oil has been demonstrated in animal models, where it has been shown to improve the redox status under certain conditions (105).

Hempseed has also been shown to induce antioxidant mediators and protein genes that protect cells from oxidative stress injury, including the induction of HO-1, an inducible enzyme that degrades haeme and produces bilirubin, a strong antioxidant (78, 106). The induction of HO-1 has been shown to protect against endothelial dysfunction and oxidative stress. It is proposed that this process may underlie a possible mechanism by which hempseed phenylpropionamides act as a protective agent (106, 107). A study on hyperhomocysteinemic rats found that hempseed supplementation improved endothelial function and reduced oxidative stress (24), whereas another study found that hempseed extract inhibited the formation of ROS and improved endothelial function in human umbilical vein endothelial cells (106).

The anti-inflammatory and antioxidant properties of hempseed oil suggest its potential therapeutic applications for reducing the effects of ischaemia-reperfusion injury. In preclinical models, hempseed oil supplementation has demonstrated multifaceted cardioprotective effects. Wijerathne et al. (108) reported a significant reduction in oxidative stress markers and inflammatory cytokines in a rat model of ischaemia-reperfusion injury following hempseed oil administration. These findings were complemented by Terada et al. (109), who observed decreased apoptotic cell death and improved myocardial contractility in rats with experimentally induced myocardial infarction treated with hempseed oil. Further supporting these cardioprotective mechanisms, Fibbi et al. (110) demonstrated that hempseed oil intervention in a heart failure model attenuated pathological cardiac remodeling while simultaneously enhancing ventricular function parameters. Collectively, these preclinical studies suggest that the bioactive components of hempseed oil may confer protection against myocardial injury by modulating oxidative and inflammatory pathways, warranting further investigation in clinical settings.

### 4.3 Lipid profile modulation

Hempseed has been found to have beneficial effects on cholesterol levels and overall lipid profiles. Studies have shown that hempseed supplementation can reduce dietary cholesterol uptake in the presence of an enriched cholesterol diet (24). In a previous study, hempseed lipid fractions reduced atherosclerotic plaque formation and improved the blood lipid profile in rats fed a high-fat diet (111). Another study found that hempseed oil decreased plasma total cholesterol and triglyceride levels and increased HDL-cholesterol levels in hyperlipidaemic mice (112). Regarding the specific mechanisms by which hempseed exerts its effects on cholesterol levels and lipid profiles, several key compounds have been identified, including PUFAs, such as LA and ALA, and phytosterols, such as beta-sitosterol (24, 79, 113, 114). These compounds have been found to downregulate the expression of the Niemann-Pick C1-Like 1 (NPC1L1) gene, which encodes a key intestinal cholesterol absorption protein (24), and to inhibit the absorption of cholesterol in the intestine (114).

Hempseed has been found to have anti-inflammatory and antioxidant properties, which can also contribute to its beneficial effects on lipid profiles (79, 113). However, it is worth noting that the effects of hempseed on cholesterol levels and lipid profiles can vary depending on the specific study and type of hempseed used. A study found that hempseed oil decreased the concentration of HDL and triglycerides but did not affect total cholesterol levels in obese male Zucker rats (115). Rupasinghe et al. (26) found that daily supplementation of 30 mL of hempseed oil for 4 weeks reduced the total plasma triglycerides (TG) concentration, significantly decreased the TC/HDL-C ratio, lowering the risk of coronary heart disease, and increased the LA, ALA, and GLA amounts in the serum triglycerides and cholesteryl esters. However, the study did not find any significant changes in plasma glucose, insulin, or haemostatic factors, such as fibrinogen, coagulation factor VIIa (FVIIa), and plasminogen activator inhibitor-1 (PAI-1). Despite these promising findings, it should be acknowledged that effects on lipid profiles appear inconsistent across studies, with some research showing minimal or no significant changes in certain lipid parameters, highlighting the need for standardized dosing and study protocols. Further research is needed to fully understand the effects of hempseed on cholesterol levels and lipid profiles in different populations under different conditions.

### 4.4 Blood pressure regulation

Hempseed plays a potential role in blood pressure regulation through various mechanisms. Bioactive peptides derived from hempseed proteins have antihypertensive properties, including the inhibition of renin and angiotensin-converting enzyme (ACE) activities (24, 28, 116, 117). Samsamikor et al. (117) conducted a randomized clinical trial comparing hemp seed protein and its hydrolysates with casein in adults with hypertension. This study found hempseed peptides could effectively modulate systolic and diastolic blood pressure and plasma biomarkers, providing direct evidence of benefit in human cardiovascular risk management. These peptides can also increase NO levels, a potent vasodilator, and reduce oxidative stress, which can contribute to the development of hypertension (28, 83). Studies have also shown that the administration of hempseed protein hydrolysates (HPH) to spontaneously hypertensive rats (SHRs) lowers systolic blood pressure (SBP) and reduces plasma ACE activity (24, 83, 116, 118, 119). HPHs have a hypotensive effect, with 1% alcalase HPH being the most effective in lowering SBP (118). Furthermore, bioactive peptides derived from hempseed proteins may play a role in regulating the renin-angiotensin-aldosterone system (RAAS) (28, 83, 119, 120).

Hempseeds contain magnesium and potassium, which promote vasodilation. Mg plays a crucial role in vasodilation, particularly in regulating endothelial function. It acts as a calcium antagonist, inhibiting the depolarization effect of calcium and the excitation-contraction coupling related to calcium, which results in the attenuation of thoracic aorta contraction and increased vasodilation at high magnesium concentrations (121). In addition to its role in vasodilation, magnesium improves endothelial function and reduces inflammation, as shown in various studies (122–124). Mg supplementation has been found to improve endothelial function in patients with coronary artery disease, heart failure, and diabetes mellitus (125, 126).

Potassium, an essential mineral found in hempseeds, also contributes to vasodilation. Potassium ions promote the relaxation of smooth muscle cells in blood vessels, facilitating vasodilation (127). Overall, the combination of magnesium and potassium in hempseeds makes them a potential natural source of vasodilation, which can be beneficial for cardiovascular health (128).

### 4.5 Vascular function improvement

Hesperidin, a flavonoid found in hempseed, has been shown to have vasoprotective properties (129). Hesperidin can improve endothelial function by increasing NO production and bioavailability (130). Additionally, hesperidin has been shown to reduce oxidative stress and inflammation in blood vessels and to protect against endothelial damage and vascular inflammation (129). A study on rabbits found that dietary hempseed supplementation did not protect against atherosclerotic plaque formation but provided mildly beneficial effects against contractile dysfunction associated with atherosclerotic vessels (24). Al-Khalifa et al. demonstrated in animal models that hempseed supplementation could significantly improve post-ischemic cardiac recovery compared to controls (131). Another study in rats found that 10% hempseed dietary supplementation improved post-ovariectomy complications, including the prevention of bone loss and dyslipidemia (24). Hempseed also contains gamma-tocopherol, a form of vitamin E that has been shown to have antioxidant and anti-inflammatory effects (132). A study on laying hens found that hempseed supplementation increased the amount of *γ*-tocopherol in egg yolks, which may have positive effects on vascular function (132).

### 4.6 Platelet aggregation and thrombosis

Animal studies have reported inconsistent findings regarding the effects of hempseed on platelet aggregation and thrombosis (112, 133–135). Some studies have suggested that hempseed supplementation can have beneficial effects on platelet aggregation, while others have found no significant effects or even potential negative effects. Lee et al. found that dietary hempseed supplementation downregulated the expression of the NPC1L1 gene, leading to reduced cholesterol uptake and platelet aggregation in *Drosophila melanogaster* (112). Similarly, Prociuk et al. showed that 8 weeks of 10% hempseed supplementation of a cholesterol-enriched diet in albino New Zealand rabbits led to an approximately 12-fold increase in plasma GLA levels, resulting in the normalization of platelet aggregation (133). However, Gavel et al. suggested that the addition of 10% hempseed to the cholesterol-enriched diet of albino New Zealand rabbits had no effect on atherogenesis or aortic contractile function (134). In contrast, studies have shown that hemin, a component of hempseed, can induce platelet activation and thrombosis. Hemin has been shown to bind to Toll-like receptor (TLR) TLR4 on endothelial cells and result in endothelial cell activation and secretion of Weibel-Palade bodies (135). Additionally, hemin can induce platelet activation via C-type lectin-like receptor-2 (CLEC-2) in both human and mouse platelets (136). Platelet activation by hemin is associated with increased P-selectin expression, GPIIb/IIIa activation, and phosphatidylserine exposure (136). Overall, the effects of hempseed on platelet aggregation and thrombosis appear to be complex and dependent on various factors, including the concentration and composition of hempseed, the animal model used, and the specific mechanisms involved.

In summary, hempseed is rich in key nutrients and bioactive compounds, such as ALA, phytosterols, and vitamin E, which contribute to cardiovascular health by reducing inflammation, lowering cholesterol absorption, and enhancing vascular function (Table 1).

**Table 1:** CVD prevention effects of key nutrients and bioactive compounds in hempseed.

Nutrient category	Specific nutrient/Compound	CVD prevention effects
Macronutrients	Alpha-Linoleic Acid	↓ Inflammation, ↓ Cholesterol Absorption
	Oleic Acid	↓ Inflammation
	Linoleic Acid	↓ Cholesterol Absorption
Micronutrients	Magnesium	↓ Inflammation, ↑ Vasodilation
	Potassium	↑ Vasodilation
	Vitamin E	↓ Inflammation, ↓ Oxidative Stress, ↑ Vascular Function
Bioactive components	Lignanamides	↓ Inflammation, ↓ Oxidative Stress
	Terpenes	↓ Inflammation
	Glaucocalyxin A	↓ Inflammation
	Phytosterols	↓ Cholesterol Absorption
	Bioactive Peptides	↓ Blood Pressure
	Hesperidin	↑ Vascular Function

## 5 Comparison with other seeds

Flaxseed (*Linum usitatissimum*) and chia seed (*Salvia hispanica* L.) have gained significant attention as functional foods based on their substantial omega-3 fatty acid content, dietary fiber, and bioactive compounds with documented cardiovascular health benefits. Hempseed and flaxseed are rich sources of PUFAs, particularly omega-3 and omega-6 fatty acids, which have potential cardiovascular benefits (131, 137). Flaxseed has a higher antioxidant capacity than hempseed, and both seeds are good sources of fiber and lipids (138). Dietary hempseed intake has shown cardiovascular benefits in animal studies, improving post-ischemic heart performance (131). However, a 12-week human study found no significant changes in cardiovascular parameters following supplementation with hempseed, flaxseed, or fish oil (137). Similarly, another research demonstrated that the impact of hempseed oil and flaxseed oil on serum lipid profiles varied, showing only modest effects on fasting serum total or lipoprotein lipids, with no meaningful alterations in plasma glucose, insulin, or haemostatic markers (139). Both hempseed and flaxseed contain EFAs, with hempseed being rich in LA and ALA (131). It should be noted that direct head-to-head clinical comparisons of these seeds’ cardiovascular effects are limited in the literature, and most comparative assessments rely on indirect comparisons of their individual study outcomes. Although these seeds offer potential health benefits, further research is needed to fully understand their lipid composition and bioactivity (140).

In contrast, hempseed and chia seeds offer distinct nutritional profiles and potential cardiovascular benefits. Hempseed is rich in omega-3 and omega-6 fatty acids, which may improve cardiovascular health (141). Both seeds demonstrate antioxidant properties, with hempseed containing higher total phenolic and flavonoid contents (142). These findings suggest that both hempseed and chia seeds may offer unique nutritional advantages and potential cardiovascular benefits. Table 2 presents a detailed nutritional comparison of hempseed, flaxseed, and chia seed, including macronutrients, lipids, essential amino acids, vitamins, and minerals. While these nutritional differences suggest potential variations in cardiovascular impact, direct comparative clinical trials examining the relative efficacy of these seeds on cardiovascular outcomes are needed to validate these theoretical advantages.

**Table 2:** Nutrient profile of hempseed, flaxseed and chia seed* (21, 24, 26–29, 31, 32, 137, 141, 142, 150, 168).

Nutrient	Units	Hempseed	Flaxseed	Chia seed
Macro				
Energy	kcal	567	534	486
Protein	g	24.8	18.3	16.5
Total Fat	g	48.8	42.2	30.7
Ash	g	5.6	3.72	4.8
Carbohydrates	g	27.6	28.9	42.1
Total fiber	g	27.6	27.3	34.4
Lipids				
Saturated	g	4.6	3.66	3.33
Monounsaturated	g	5.4	7.53	2.31
Polyunsaturated	g	38.1	28.7	23.7
LA	g	27.4	5,9	5.84
GLA	g	4	n/a	n/a
ALA	g	8.68	22.81	17.8
Cholesterol	g	0	0	0
Essential Aminoacids				
Histidine	g	0.71	0.472	0.531
Isoleucine	g	0.98	0.896	0.801
Leucine	g	1.72	1.24	1.37
Lysine	g	1.03	0.862	0.97
Methionine	g	0.58	0.37	0.588
Phenylalanine	g	1.17	0.957	1.02
Threonine	g	0.88	0.766	0.709
Tryptophan	g	0.20	0.297	0.436
Valine	g	1.28	1.07	0.95
Vitamins				
Vitamin C	mg	1	0.6	1.6
Thiamin	mg	0.4	1.64	0.62
Riboflavin	mg	0.11	0.161	0.17
Niacin	mg	2.8	3.08	8.83
Vitamin B6	mg	0.12	0.473	0.393
Vitamin A	IU	3,800	0	54
Vitamin D	IU	2277.5	0	0
Vitamin E	mg	90	0.31	0.5
Minerals				
Calcium (Ca)	mg	145	255	631
Iron (Fe)	mg	14	5.73	7.72
Magnesium (Mg)	mg	483	392	335
Phosphorus (P)	mg	1,160	642	860
Potassium (K)	mg	859	813	407
Sodium (Na)	mg	12	30	16
Zinc (Zn)	mg	7	4.34	4.58
Copper (Cu)	mg	2	1.22	0.924
Manganese (Mn)	mg	7	2.48	2.72
Selenium (Se)	mcg	0.02	25.4	55.2

^*^All values represent nutrient content per 100 g of seed. Units are as specified in the “Units” column (g = grams, mg = milli-grams, mcg = micrograms, IU = International Units, kcal = kilocalories). n/a = not applicable.

Hempseed exhibits the highest energy density (567 kcal) and protein content (24.8 g) among the three seeds, making it an excellent nutritional source for maintaining a healthy body composition and muscle mass, which are increasingly recognized in cardiovascular health maintenance. Additionally, Hempseed demonstrates a superior essential amino acid profile with higher levels of isoleucine, leucine, lysine, and valine than either flaxseed or chia seed. This comprehensive amino acid composition supports vascular integrity and may contribute to improved endothelial function, a critical factor in CVD prevention (143, 144).

The PUFA content of hempseed (36.2 g) substantially exceeded those of both flaxseed (28.7 g) and chia seed (23.7 g). Most remarkably, hempseed contains 56 g of LA, which is approximately ten times the amount found in flaxseed (5.9 g) and chia seed (5.84 g). Additionally, hempseed uniquely provides GLA (4 g) alongside balanced EFAs, which are absent in other seeds and have been associated with anti-inflammatory effects relevant to atherosclerosis prevention (21, 111, 141).

Although all three seeds contain significant ALA, with hempseed and flaxseed providing comparable amounts (22 g and 22.81 g, respectively), the balanced omega-6 to omega-3 ratio in hempseed may confer optimal inflammatory modulation effects that could mitigate cardiovascular risk factors (111, 141).

The fiber content of these seeds further supports cardiovascular health by improving lipid metabolism and reducing blood cholesterol levels. Chia seed contains the highest total fiber (34.4 g/100 g), followed by hempseed (27.6 g) and flaxseed (27.3 g). However, the fiber in hempseed consists of both soluble and insoluble fractions, aiding in cholesterol regulation and gut microbiota balance, both of which are critical for cardiovascular health (115, 145, 146).

From the micronutrient perspective, hempseed is particularly rich in magnesium (483 mg), phosphorus (1,160 mg), potassium (859 mg), and iron (14 mg), all of which play essential roles in vascular function, blood pressure regulation, and redox balance. In addition, hempseed contains the highest concentrations of zinc (7 mg), copper (2 mg), and manganese (7 mg), which are involved in antioxidant defense systems and metabolic processes relevant to cardiovascular health (147). Moreover, hempseed contains dramatically higher levels of vitamin E than flaxseed (0.31 mg) or chia seed (0.5 mg), providing potent antioxidant protection against lipid peroxidation and endothelial dysfunction (54, 56, 58, 148). Additionally, Hempseed is the only seed among the three that contains significant amounts of vitamin D (2277.5 IU) and substantial amounts of vitamin A (3,800 IU), both of which are implicated in cardiovascular health through anti-inflammatory and immunomodulatory pathways (45). Compared with other functional seeds commonly associated with cardiovascular benefits, hempseed exhibits a distinctive profile of bioactive compounds that may confer unique cardioprotective properties (Table 3).

**Table 3:** Comparative bioactive compound profile of hempseed, flaxseed, and chia seed in relation to cardiovascular benefits (140, 141, 150, 168).

Bioactive component	Hempseed	Flaxseed	Chia seed
γ-Tocopherol (mg/100 g)	60–80	10–15	5–7
Total Phytosterols (mg/100 g)	420–670	250–300	350–400
Major Phytosterol	β-sitosterol	β-sitosterol	β-sitosterol
Total Phenolics (mg GAE/g)	2.2–3.7	8.0–10.5	6.5–8.8
Major Phenolic Compounds	Phenolic acids	Lignans	Flavonoids
Unique Components	Lignanamides	S.diglucoside	Caffeic acid derivatives

While flaxseed contains higher concentrations of phenolic compounds, particularly lignans with demonstrated oestrogenic and antioxidant properties, hempseed has substantially higher *γ*-tocopherol content. This distinction is noteworthy, given the growing evidence of the superior anti-inflammatory properties of γ-tocopherol in vascular tissues compared to other tocopherol isomers (148, 149).

Similarly, although chia seeds contain a richer flavonoid profile, hempseed provides a variety of phytosterols, which may offer more significant cholesterol-lowering benefits. The unique lignanamide compounds found in hempseed, including cannabisin derivatives, represent another distinguishing feature with potential cardiovascular implications that require further investigation.

While hempseed demonstrates certain nutritional advantages over flaxseed and chia seeds, it is worth noting that other seeds may provide superior benefits in specific areas (150), such as flaxseed’s higher lignan content and chia seed’s greater fiber density, suggesting that no single seed provides optimal cardiovascular protection alone (23, 24, 150). Future research should prioritize randomized controlled trials directly comparing these functional seeds to establish evidence-based recommendations for cardiovascular health, rather than relying on indirect nutritional profile comparisons.

This comparison emphasizes that, while each functional seed offers distinct bioactive profiles and potential health benefits, hempseed contributes a unique constellation of bioactive compounds to the dietary resource against CVD. Therefore, integration of hempseed with other functional seeds in dietary recommendations may provide complementary bioactive compounds for comprehensive cardiovascular protection.

## 6 Safety and considerations

Despite its botanical relationship with psychotropic cannabis varieties, hempseed derived from industrial hemp (*Cannabis sativa* L.) contains negligible *Δ*-9 THC content and has gained attention for its potential cardiovascular benefits, although safety considerations and regulatory aspects require careful evaluation (141). Legal restrictions on conventional hemp have been addressed by developing cultivars with minimal THC levels. Hempseed cultivars containing less than 0.3% THC, classified as non-drug varieties, have attracted considerable attention for their nutritional and therapeutic applications (26, 27). Although hemp-based food products generally have low safety concerns, some products, such as hemp seed oils, may pose a moderate risk because of their variable THC content.

### 6.1 Allergenic potential

An important safety consideration is the allergenic potential of hempseed consumption. Emerging evidence suggests that certain proteins within hempseed may trigger allergic reactions in susceptible individuals with pre-existing sensitivities to plant-based proteins. Key allergens, such as thaumatin-like proteins and edestin, have been identified as potential triggers (151). Although the overall prevalence of hempseed allergy remains poorly documented, cross-reactivity with other allergenic seeds and nuts has been reported, highlighting the need for further epidemiological research.

Post-harvest processing of hempseed for nutraceutical and nutritional applications not only enhances nutrient bioavailability and palatability but also mitigates the presence of allergens (152). For example, the pre-digestion of hempseed proteins (HSPs) using intestinal brush border hydrolytic enzymes has been shown to substantially reduce allergen levels, including thaumatin-like proteins (151). This indicates that enzymatic hydrolysis effectively degrades allergenic epitope-specific amino acid sequences that trigger allergic reactions. Furthermore, the thermal processing and proteolysis employed in the production of hempseed flour for commercial food products can reduce its allergenicity (151). However, additional research is necessary to ensure that such processing methods do not compromise the stability or health-promoting properties of HSPs and their derived peptides.

### 6.2 Regulatory status and quality control

The legal and regulatory status of hempseed varies considerably across countries owing to its association with *Cannabis sativa* L. Despite having negligible levels of THC, hempseed classification and acceptance as a food product remain subject to stringent regulatory frameworks worldwide.

In the United States and Canada, federal regulations mandate that hempseed products contain no more than 0.3% THC on a dry weight basis. Similarly, the European Union has set a THC threshold of 0.2% for hemp cultivation and consumption, although some member states have enforced stricter limits on THC levels. Recent policy changes in regions such as Australia, Canada, and the United States have led to the legalization of low-THC hemp varieties for food use (23).

Regulatory compliance remains a challenge, as studies have found that THC concentrations in some consumer-grade hemp seeds exceed legal limits by up to 1,250% (153). In Germany, a significant decrease in THC concentrations in hemp products was observed between 1998 and 2003, which was attributed to EU regulations mandating the use of certified hempseed (154). Despite these improvements, marketing hemp-derived products remains subject to complex regulations, partly due to the presence of cannabidiol (CBD) in pharmaceutical agents and varying policies on cannabis inflorescence (27).

A key challenge in harmonizing global regulations lies in the lack of consensus on acceptable THC limits and potential cross-contamination during cultivation and processing. To address these disparities, international organizations such as the Codex Alimentarius Commission and the World Health Organization (WHO) have begun developing guidelines for hempseed safety and quality. However, the application of these guidelines remains inconsistent, emphasizing the need for ongoing discussions and evidence-based informed policy decisions.

#### 6.2.1 Interactions with anticoagulants

Hempseed is a notable source of omega-3 and omega-6 fatty acids, particularly ALA and GLA, which have been shown to exert antithrombotic effects by modulating platelet aggregation and reducing blood viscosity. Although beneficial for CVD prevention, these properties pose a potential risk to patients receiving anticoagulant therapy. Concurrent consumption of hempseed and anticoagulants may enhance the risk of bleeding due to their additive effects on coagulation pathways, as omega-3 fatty acids can inhibit the synthesis of thromboxane A2, a pro-thrombotic eicosanoid.

Recent studies have explored the potential interactions between cannabinoids and anticoagulants, with a focus on warfarin. Case reports suggest that high doses of cannabis may increase the effects of warfarin, potentially leading to elevated international normalized ratio (INR) levels and increased bleeding risk (155, 156). Cannabis components can inhibit or compete with cytochrome P450 enzymes, potentially affecting the metabolism of anticoagulants and antiplatelet agents (156). Although direct clinical evidence specific to hempseed is limited, caution is warranted, particularly in individuals undergoing chronic anticoagulation therapy (157).

#### 6.2.2 Interactions with antihypertensive medications

Hempseed contains high levels of arginine, a precursor of NO, which plays a crucial role in vasodilation and endothelial function. In addition, hempseed-derived peptides have demonstrated ACE inhibitory activity. When combined with antihypertensive medications, such as ACE inhibitors, calcium channel blockers, or beta-blockers, the vasodilatory and ACE-inhibitory effects of hempseed may potentiate the action of these drugs, leading to excessive reductions in blood pressure. This can result in hypotensive symptoms, particularly in individuals with well-controlled hypertension. Therefore, patients with hypertension should monitor their blood pressure regularly and consult healthcare providers before significantly increasing their intake of hempseed or hempseed-based products (158).

### 6.3 Recommendations for safe consumption

Given the growing interest in hempseed as a functional food with potential cardiovascular benefits, healthcare providers should consider potential food-drug interactions when recommending it as part of a heart-healthy diet. Certain populations may be at increased risk for adverse effects from hempseed consumption (159). Pregnant and lactating women should be cautious due to limited safety data in these populations. Individuals with severe cardiovascular disease, particularly those with unstable angina or recent myocardial infarction, should consult healthcare providers before incorporating hempseed supplements, as the vasodilatory effects may interact with existing cardiovascular instability. Patients with bleeding disorders or those scheduled for surgery should discontinue hempseed consumption at least 2 weeks prior to procedures due to potential anticoagulant effects (156, 160). Although moderate consumption is unlikely to cause significant adverse effects, individuals on anticoagulant or antihypertensive therapy should consult their healthcare providers before increasing their intake.

To fully utilize the health-promoting properties of hempseed in food products, it is essential to ensure their safety through comprehensive toxicity and allergenicity evaluations. Given the increasing incorporation of hempseed-derived ingredients into functional foods, continued risk assessment is essential to ensure consumer safety, particularly in individuals with existing seed or nut allergies.

The food industry’s exploration of hempseed as both a livestock feed supplement and an ingredient to enrich daily foods necessitates more comprehensive investigations to fully understand the role of hempseed in functional foods and human health (24). Future research is needed to explore the clinical significance of potential drug interactions and establish evidence-based dietary guidelines that optimize the cardioprotective benefits of hempseed while minimizing potential risks.

## 7 Potential applications in diet

Hempseed is a nutrient-dense functional food that can be seamlessly integrated into diverse dietary patterns worldwide. Its mild, nutty flavor and rich nutritional profile make it a versatile ingredient across various culinary traditions. Given the increasing global emphasis on plant-based nutrition and sustainable food sources, incorporating hempseeds into daily meals offers an innovative approach to enhance dietary quality. Its integration into both traditional and modern food systems represents a promising strategy for promoting balanced, nutrient-dense diets worldwide. For example, in Western dietary patterns, which are characterized by a high intake of processed foods and animal-based products (161), hempseed serves as a valuable supplement to both plant-based and omnivorous diets. It can be incorporated into breakfast cereals, smoothie bowls, and yoghurt products to enhance protein and omega-3 fatty acid intake. Additionally, hempseed flour can be used in baking bread, muffins, and protein bars, offering a gluten-free alternative with improved nutritional value. Similarly, hempseed can complement the Mediterranean dietary pattern by being included in traditional dishes, such as hummus, pesto, and tzatziki, where it enhances the protein content and provides EFAs. Hempseed can also be incorporated into grain-based salads, such as tabbouleh, or added to roasted vegetable dishes to enrich their nutritional profile. In East Asian diets, hempseed can be blended into congee (rice porridge), sprinkled over steamed vegetables, or used as a topping in noodle dishes. In South Asian cuisine, where lentils and legumes form dietary staples (162), hempseed can be incorporated into dals, chutneys, or spiced yoghurt-based side dishes, such as raita. Moreover, in Southeast Asian culinary traditions, hempseed can be included in coconut-based curries or blended with plant-based milk alternatives.

The integration of hempseed into daily dietary practices faces several cultural and regulatory challenges (24, 163). Consumer acceptance remains limited due to persistent associations with psychoactive cannabis, despite hempseed’s negligible THC content (23, 164). Educational initiatives emphasizing the distinction between industrial hemp and marijuana varieties are essential for broader acceptance. Public health strategies must address these cultural stigmas through evidence-based messaging while working with regulatory bodies to establish consistent, science-based guidelines for hempseed products in the food supply.

Hempseed has garnered attention in the formulation of nutraceutical products owing to its rich content of omega-3 and omega-6 fatty acids, high-quality protein, and bioactive compounds (32). Hemp protein powders and snack bars fortified with hempseed offer plant-based alternatives to traditional protein sources, appealing to the growing demand for sustainable and functional foods. Beyond stand-alone products, hempseed has been incorporated into dairy and meat alternatives such as, hemp milk and plant-based burger patties, providing both nutritional and sensory benefits. Functional beverages, including hemp-infused teas and smoothies, have emerged as potential delivery systems for bioactive compounds that support cardiovascular health. Further research on the role of hempseed in cardiovascular health will be instrumental in optimizing its use in targeted food formulations.

## 8 Study limitations and evidence quality

The current evidence base for hempseed’s cardiovascular benefits is predominantly derived from preclinical studies, which present significant limitations in translating findings to human health outcomes. Animal models, while significant for mechanistic perception, may not accurately reflect human cardiovascular physiology due to species-specific differences in metabolism, lipid handling, and inflammatory responses. The extrapolation of dosages from animal studies to humans is a particular issue (165, 166), as the bioavailability and metabolic pathways of hempseed bioactive compounds may differ substantially between species.

Furthermore, the limited human clinical trials available exhibit considerable methodological heterogeneity, including variations in study populations, hempseed preparations, dosing regimens, and outcome measurements. Many studies lack adequate statistical power, randomization, or control groups, limiting the reliability of their conclusions. The short-term nature of most human studies (typically 4–12 weeks) fails to capture long-term cardiovascular outcomes or potential adverse effects of chronic hempseed consumption.

## 9 Gaps in knowledge and future directions

Despite a growing body of evidence supporting the potential of hempseed in CVD/CMD prevention, several critical research gaps remain that inhibit its broader acceptance and application in clinical and public health settings. While short-term studies have demonstrated the potential effects of hempseed on lipid profiles, inflammation, and oxidative stress (137, 139), there is a lack of long-term randomized controlled trials (RCTs) assessing its sustained impact on CVD/CMD outcomes. It is important to emphasize that the majority of mechanistic evidence comes from *in vitro* and animal studies, which may not accurately predict clinical outcomes in diverse human populations with varying baseline health status and genetic backgrounds (165, 167). Long-term data are essential to understand the durability of the benefits of hempseed and to identify any potential adverse effects associated with prolonged consumption. In addition, the majority of existing studies are small-scale or conducted in specific populations, and the generalizability of the findings is limited. Large-scale epidemiological studies are needed to establish the relationship between hempseed consumption and CVD/CMD risk across diverse populations, including different age groups, ethnicities, and individuals with varying baseline health statuses. Moreover, the optimal dose of hempseed or its derivatives (e.g., hempseed oil and protein) for CVD/CMD prevention has not been established. Further studies are needed to determine the minimum effective dose, potential upper limits, and whether synergistic effects exist when combined with other dietary components. Furthermore, data on the effects of hempseed in high-risk populations such as, individuals with diabetes, metabolic syndrome, or established CVD, are limited and often inconclusive. Research focusing on these subgroups is critical for tailoring dietary recommendations and assessing the therapeutic potential of hempseed in reducing the progression of diseases.

To address these gaps and elevate the status of hempseed as a functional food for CVD/CMD prevention, well-designed, long-term RCTs should be conducted to evaluate the effects of hempseed consumption on hard CVD endpoints (e.g., myocardial infarction, stroke, and mortality) and intermediate markers (e.g., blood pressure, arterial stiffness, and inflammatory biomarkers). These studies should include diverse populations and incorporate rigorous dietary controls to isolate the effects of hempseed. Large-scale prospective cohort studies are needed to examine the association between habitual hempseed intake and CVD/CMD incidence. The dose–response relationships of hempseed and its derivatives should be investigated to identify optimal doses for specific health outcomes. In addition, the development of standardized formulations with enhanced bioavailability to maximize therapeutic efficacy should be explored. Collaborations among nutrition scientists, cardiologists, food technologists, and public health experts should be established to design comprehensive studies that integrate clinical, biochemical, and dietary perspectives. Such interdisciplinary approaches enhance the robustness and translational relevance of the findings.

## 10 Conclusion

In conclusion, hempseed represents a highly promising functional food with significant potential for CVD prevention. Its rich nutritional composition, such as high-quality plant proteins, EFAs, and a variety of bioactive compounds, positions it as a valuable dietary component for improving cardiometabolic health. This review emphasizes several key mechanisms through which hempseed exerts cardioprotective effects, including its ability to modulate lipid profiles, reduce inflammation and oxidative stress, and improve vascular function. These effects are largely attributed to its unique bioactive components, including tocopherols, phytosterols, and polyphenols, which collectively contribute to its anti-inflammatory and antioxidant properties. When comparing hempseed, flaxseed, and chia seed, hempseed might be the most nutritionally balanced option for cardiovascular support. It is crucial to acknowledge that current evidence predominantly relies on animal studies and small-scale human trials, with several studies showing mixed or non-significant results, indicating that the cardiovascular benefits of hempseed may be more modest than initially suggested by early preclinical research.

Future research should prioritize well-designed, multi-center RCTs with specific design features to address current evidence gaps. Priority studies should include: (1) dose–response trials of daily hempseed intake over a wide range of periods in adults, measuring hard endpoints including myocardial infarction and stroke incidence; (2) mechanistic studies using advanced imaging (coronary CT angiography, flow-mediated dilation) to evaluate hempseed’s effects on atherosclerotic plaque progression and endothelial function; (3) pharmacokinetic studies examining bioactive compound absorption and metabolism to optimize formulation and timing of intake; and (4) head-to-head comparisons with established cardioprotective interventions (statins, omega-3 supplements) in high-risk populations. Pragmatic effectiveness trials within existing cohort studies could help evaluate how hempseed fits into dietary patterns across diverse populations. At the same time, safety surveillance studies are needed to monitor potential drug interactions in patients taking anticoagulant or antihypertensive therapy.

The integration of hempseed into various dietary patterns worldwide offers a versatile and sustainable approach to enhance dietary quality and promote cardiovascular health. This review clarifies the importance of continued interdisciplinary collaboration among nutrition scientists, cardiologists, food technologists, and public health experts to advance our understanding of the role of hempseed in CVD prevention and promote its integration into global dietary recommendations.

## Glossary

AA: Arachidonic acid
ACE: Angiotensin-converting enzyme
ALA: *α*-linolenic acid
CB2: Cannabinoid receptors type 2
CLEC-2: C-type lectin-like receptor-2
CLG: Coumaroylaminobutanol glucopyranoside
CMD: Cardiometabolic disorders
COX-2: Cyclooxygenase-2
CRP: C-reactive protein
CVD: Cardiovascular disease
DHA: Docosahexaenoic acid
DPPH: 2,2-diphenyl-1-picrylhydrazyl
DRI: Dietary Reference Intake
DRV: Dietary Reference Value
EFA: Essential fatty acid
EPA: Eicosapentaenoic acid
FVIIa: Fibrinogen, coagulation factor VIIa
GAE: Gallic acid equivalents
GLA: *γ*-linolenic acid
HO-1: Haeme oxygenase-1
HPH: Hempseed protein hydrolysates
ICAM-1: Intercellular adhesion molecule 1
IL-6: interleukin6
IL-1β: interleukin-1 beta
iNOS: inducible nitric oxide synthase
LDL: Low-density lipoprotein
LPS: Lipopolysaccharide
MMPS: Matrix metalloproteinases
NF-κB: Nuclear factor kappa B
Nfr-2: Nuclear factor erythroid 2-related factor 2
NO: Nitric oxide
NPC1L1: Niemann-Pick C1-Like 1
ORAC: Oxygen radical absorbance capacity
PAI-1: Plasminogen activator inhibitor-1
PUFA: Polyunsaturated fatty acid
RCT: Randomized controlled trial
ROS: Reactive oxygen species
SA: Stearic acid
SBP: Systolic blood pressure
SDA: Stearidonic acid
SFA: Saturated fatty acid
T2DM: Type 2 diabetes mellitus
TE: Trolox equivalents
TG: Triglycerides
THC: Tetrahydrocannabinol
TNF: Tumor necrosis factor
VCAM-1: Vascular cell adhesion molecule 1
WHO: World Health Organisation
